# Metabolomic profiles and microbiota of GDM offspring: The key for future perspective?

**DOI:** 10.3389/fped.2022.941800

**Published:** 2022-10-05

**Authors:** Angelica Dessì, Chiara Tognazzi, Alice Bosco, Roberta Pintus, Vassilios Fanos

**Affiliations:** Neonatal Intensive Care Unit, Department of Surgical Sciences, Azienda Ospedaliera Universitaria (AOU) Cagliari, University of Cagliari, Cagliari, Italy

**Keywords:** GDM, metabolomics, microbiota, microbiome, obesity

## Abstract

Gestational diabetes mellitus (GDM), or any degree of glucose intolerance recognized for the first time during pregnancy, is one of the diseases that most frequently aggravates the course of gestation. Missed or late diagnosis and inadequate treatment are associated with high maternal and fetal morbidity, with possible short- and long-term repercussions. Estimates on the prevalence of GDM are alarming and increasing by about 30% in the last 10–20 years. In addition, there is the negative influence of the SARS-CoV-2 emergency on the glycemic control of pregnant women, making the matter increasingly topical. To date, knowledge on the metabolic maturation of newborns is still incomplete. However, in light of the considerable progress of the theory of “developmental origins of health and disease,” the relevant role of the intrauterine environment cannot be overlooked. In fact, due to the high plasticity of the early stages of development, some detrimental metabolic alterations during fetal growth, including maternal hyperglycemia, are associated with a higher incidence of chronic diseases in adult life. In this context, metabolomic analysis which allows to obtain a detailed phenotypic portrait through the dynamic detection of all metabolites in cells, tissues and different biological fluids could be very useful for the early diagnosis and prevention of complications. Indeed, if the diagnostic timing is optimized through the identification of specific metabolites, the detailed understanding of the altered metabolic pathway could also allow better management and more careful monitoring, also from a nutritional profile, of the more fragile children. In this context, a further contribution derives from the analysis of the intestinal microbiota, the main responsible for the fecal metabolome, given its alteration in pregnancies complicated by GDM and the possibility of transmission to offspring. The purpose of this review is to analyze the available data regarding the alterations in the metabolomic profile and microbiota of the offspring of mothers with GDM in order to highlight future prospects for reducing GDM-related complications in children of mothers affected by this disorder.

## Introduction

The American Diabetes Association defines gestational diabetes mellitus (GDM) as any degree of glucose intolerance recognized for the first time during pregnancy, mainly in the second or third trimester of gestation (1). Insulin resistance and ß-cellular dysfunction are critical factors in the pathophysiology of this metabolic alteration (2). The maternal risk factors are now well-known, they can be are modifiable and non-modifiable. The former includes overweight and pre-pregnancy maternal obesity, metabolic syndrome and polycystic ovary syndrome, previous pre-eclampsia in previous pregnancies and stress (3). There are also other non-modifiable conditions such as maternal age, the gravidity and parity, a genetic predisposition as well as a family history of hyperglycemia, ethnicity and social-economic status (3).

GDM, if not diagnosed and treated properly, is associated with high maternal and fetal morbidity, with possible repercussions throughout their life span (4). Indeed, data on the short and long-term impact on both the mother and the child are now numerous (2, 5). Estimates of the prevalence of glycemic changes in pregnancy are not encouraging. In fact, it is believed that, in 2017, about 21.3 million live birth, or 16.2% of them, were affected by some form of maternal hyperglycemia and that 18.4 millions of them are precisely caused by GDM (about the 86.4% of pregnancy hyperglycemia) (6). The 9th edition of the IDF Diabetes Atlas released in 2019 estimated a slight reduction in the overall prevalence of maternal hyperglycemia to 15.8%, with GDM at 12.8% (7).

These data reflect an increase of this pathology of about 30% in the last 10–20 years, which affects not only industrialized countries but also developing countries (8) with variable proportions depending on the geographical area (9). Furthermore, the data available to date would seem to confirm the negative influence of the SARS-CoV-2 emergency on the glycemic control of pregnant women, making the issue increasingly topical and crucial. In fact, a worsening of diabetes control during the COVID-19 pandemic lock-down emerged from a French retrospective study (10), probably confirming the impact of reduced physical activity, changes in eating habits and associated stress. These results were reinforced by Cauldwell et al. (11) highlighting a 33% increase in the diagnosis of GDM since the beginning of the pandemic.

To date, in light of the increasing evidence of the theory of “developmental origins of health and disease” (DOHaD), the importance of the environmental impact on the phenotypic expression of an individual has been affirmed, including the intrauterine environment. It is now known that metabolic alterations during fetal growth are correlated with the occurrence of chronic diseases in adult life, such as type 2 diabetes, atherosclerosis, hypertension and metabolic syndrome, due to the high plasticity of the early stages of development (12). In this context, maternal hyperglycemia plays a very important role as a risk factor (13–15).

Metabolomics, through the analysis of a large number of low molecular weight metabolites, makes it possible to instantly identify the changes in the various metabolic pathways resulting from the interaction between specific pathophysiological states, gene expression and the environment (16). Therefore, this technique could be very useful. On the one hand, the diagnostic timing, prognosis and management of GDM could be optimized, on the other hand it would allow the improvement of the outcomes of the children of a diabetic mother. Indeed, the understanding of the specific altered metabolic pathways and the early recognition of metabolic profiles associated with the development of diseases in adult life would allow a personalized intervention even from a nutritional point of view, with a view to an individualized medicine for the most fragile children (16).

In this context, a valid contribution can also be provided by the analysis of the microbiota, the main responsible for the fecal metabolome, which has been found to be altered in pregnancies complicated by GDM, with the possibility of transmission to the offspring (17, 18).

## Epigenetics

In the scientific community, there is a growing optimism about the possibility of using the information coming from epigenetics studies as potential biomarkers to predict the probability of future adverse effects in offspring. The possibility of determining whether GDM causes an increased risk of metabolic problems in children would represent an important tool for intervention against an intergenerational cycle favoring the onset of obesity and type 2 diabetes (19). In fact, once the causal effect will be clarified, the epigenetic mechanisms could represent a possible target to prevent the negative outcomes (19). Nevertheless, the evidence from epigenetic epidemiology regarding the potential mechanism that would mediate this association is, at the moment, still not very solid (19). This is what emerged from a recent review of the scientific literature by Elliot et al. (19), which examined all human studies published in the literature regarding the association between different glycemic changes during pregnancy (including GDM and type 2 diabetes) and any epigenetic markers (DNA methylation, histone modification and miRNA). In fact, some critical issues emerged in these studies. Primarily, the small number of samples, secondly a poor replication but above all a lack of investigation of causal effects. The authors therefore conclude that further research in this area is needed with the aim to replicate what has already been observed, then the expansion of causal analysis approaches, and where possible, to investigate whether the GDM-associated epigenetic mechanisms are directly related to adverse effects in offspring even in the long term (19).

However, this analysis showed that the studies on this subject are still quite numerous and that most of them revealed alterations in DNA methylation (83% of the studies analyzed) in the cord blood, placenta and blood of the newborn (19). Overall, from the examination of the studies analyzed both by the review by Elliot et al. (19) and by that conducted in the same year period by Franzago et al. (20), it emerges that the possibility of acquiring information on the epigenetic alterations induced by an adverse intrauterine environment may represent the first step to understand the pathophysiological mechanism underlying the development of adverse effects.

An in-depth analysis of epigenetic epidemiology studies is beyond the scope of this review, nevertheless, it may be useful to understand the correlation between certain metabolic pathways and the main GDM-related epigenetic markers of some genes potentially responsible for metabolic disorders in offspring. In addition, the detection of particular epi-markings associated with glycemic changes during gestation could also be important for optimizing the interpretation of metabolomic analysis data.

Indeed, in 2013, an important impact of epigenetic labeling emerges from the study by Ruchat et al. (21). This is the first study that not only investigated the effects of GDM on the entire neonatal epigenome but also studied the possible consequences on the growth and development of the child. They showed that GDM can affect the methylation of more than 3,000 genes, both at the placental level and in the cord blood. These genes are involved in metabolic disorders as well, including diabetes, and in the regulation of energy expenditure and adiposity.

The fetal methylation state of the leptin gene (LEP) and that of adiponectin (ADIPOQ) have quite often been the subject of human epigenetics studies (20) (Table 1), due to the critical contribution of these adipokines to metabolic homeostasis (13, 22). The first study concerning the methylation of these two genes, was performed by Bouchard et al. (13, 14), in 2010. They highlighted not only the correlation between GDM and LEP methylation status, but also the difference in epigenetic labeling between the fetal and maternal side of the placenta, suggesting a different impact of maternal glycemic alterations in the placental sides resulting in LEP hyper-methylation and LEP hypo-methylation on the maternal and fetal side, respectively. In 2012 (14), they focused their attention on the methylation of ADIPOQ, highlighting a state of hypo-methylation at the level of the promoter of this gene on the fetal side of the placenta. The speculations made by the authors mainly concern the involvement of ADIPOQ and LEP in energy metabolism and in the control of insulin sensitivity. Thus, they hypothesized that these epigenetic adaptations could potentially be responsible for the metabolic disorders of the offspring over the course of life. This possible correlation is of even greater importance in light of the findings of García-Cardona et al. (23). They highlighted the presence of a negative association between methylation at the level of specific sites of LEP and ADIPOQ promoters and the combined presence of obesity, and insulin resistance, supporting the hypothesis that epigenetic modifications could be at the basis of the development of obesity and related metabolic disorders.

**Table 1:** Human studies investigating epigenetic alteration of LEP and ADIPOQ.

**References**	**Sample**	**Bio-specimens**	**Technique**	**Main findings**
Bouchard et al. (13)	48 newborns, 23 from GDM mothers	Placenta (fetal) and UCB	Bisulphite pyrosequencing	Significant correlations between LEP DNA methylation and 2-h post-OGTT glucose concentration Difference in epigenetic labeling between the fetal and maternal side of the placenta
Bouchard et al. (14)	100 newborns, 31 from GDM mothers	Placenta (fetal) and UCB	Bisulphite pyrosequencing	The ADIPOQ DNA methylation profile was associated with maternal glucose status. Lower DNA methylation levels in the promoter of ADIPOQ on the fetal side of the placenta were correlated with higher maternal glucose levels during the second trimester of pregnancy (2-h post-OGTT glucose concentration).
Ott et al. (22)	55 newborns, 25 from GDM mothers	VAT, SAT and UCB blood samples	Bisulphite pyrosequencing qRT-PCR	Variously alteration of ADIPOQ DNA methylation levels in GDM offspring cord blood cells
Gagné-Ouellet et al. (23)	259 newborns, 18 from GDM mothers	Placenta (fetal) and UCB	Infinium MethylationEPIC Beadchip	3 CpGs associated with neonatal leptinemia, cg05136031 and cg15758240, also associated with BMI and fat distribution at 3-years-old, respectively. Maternal glycemia correlated with DNA methylation at cg15758240 and neonatal leptinemia.
Gagné-Ouellet et al. (24)	60 newborns, 30 from GDM mothers	Placenta (fetal) and UCB	Infinium MethylationEPIC Beadchip	GDM was associated with changes in DNA methylation in a number of placental genes, without correlation. with observed biomarkers of metabolic health in cord blood. A trend toward a positive correlation between methylation of a CpG site in the promoter of LEP (cg05136031) and leptin in cord blood was observed.

GDM, gestational diabetes; UCB, umbilical cord blood, OGTT, Oral Glucose Tolerance Test; VAT, visceral adipose tissue; SAT, subcutaneous adipose tissue, ADIPOQ, adiponectin; LEP, leptin; CpGs, CpG islands.

While, in 2018, Ott et al. investigated the alterations of adiponectin plasma, mRNA, and DNA methylation levels in GDM offspring, showing that while newborn adiponectin levels were similar between groups (GDM and controls), DNA methylation in GDM offspring was variously altered (22).

Furthermore, Gagné-Ouellet et al. (24) investigated the association between maternal hyperglycemia, placental LEP DNA methylation, neonatal leptinemia and adiposity at 3-years-old, in 259 mother–child dyads. They observed three CpG islands associated with neonatal leptinemia, two of which, cg05136031 and cg15758240, were also associated with BMI and fat distribution at 3-years-old, respectively. Maternal glycemia was correlated with DNA methylation at cg15758240 and neonatal leptinemia. The authors speculated that DNA methylation regulation of the leptin pathway in response to maternal glycemia might be involved in programming adiposity in childhood. In addition, recently, Wang et al. (25) attempted a genome-wide identification of differentially methylated placental genes and specific GDM-associated pathways to assess the presence of correlations with fetal growth and biomarkers of metabolic health in cord blood. Results showed that although GDM was associated with changes in DNA methylation in a number of placental genes, these gene methylations were not correlated with observed biomarkers of metabolic health such as fetal growth factors, leptin and adiponectin, in cord blood. However, a trend toward a positive correlation between methylation of a CpG site in the promoter of LEP (cg05136031) and leptin in cord blood was observed, consistent with what was reported by Gagné-Ouellet et al. (24). In contrast, no correlation was observed for the cg15758240 site, unlike in the previous study. The authors conclude that the lack of significant association in the study can be partly attributed to the relatively small sample size (*n* = 60).

Epigenetic markings at the level of brown adipose tissue (BAT) have also aroused interest, given its potential preventive activity against obesity. Indeed, the findings by Côté et al. (26) suggest that the elevated maternal blood glucose levels during gestation may be responsible for changes in the DNA methylation status at the level of specific BAT-related genes, including PRDM16, BMP7 and PPARGC1α. It also emerged that some of these epigenetic variations (PRDM16, PPARGC1α) seem to influence the leptin levels in the cord blood with possible repercussions on the regulation of the body weight of the unborn child.

While, El Hajj et al. (27), focused their attention on a possible action at the level of white adipose tissue (WAT). They detected an important hypo-methylation of mesoderm-specific transcript (MEST) in placental tissues and it was found also in cord blood in the presence of GDM (both treated with insulin and diet therapy). The correlation of these results with the observation of an equally conspicuous hypo-methylation of MEST in obese adult patients has led to the hypothesis that an alteration of the epigenetic programming of this gene may contribute to the predisposition to obesity of the offspring of diabetic mother. In fact, the role of MEST as placental and fetal growth factor was highlighted in studies on knockout murine specimens (28). Furthermore, the investigation on genetically identical mice with different expression of MEST revealed distinct levels of development of diet-related obesity (29). Moreover, murine specimens in which the isoform-1 of MEST was hyper-expressed were characterized by a greater expansion of fat mass (30). However, the role and complexity of epigenetic variations in MEST are still to be investigated. Indeed, Karbiener et al. (31) suggested its role as an inhibitor of human adipogenesis in the early stages of adipocyte differentiation, unlike what was found in previous murine studies (32–34). Nevertheless, the positive correlation between MEST and fat mass was confirmed and higher levels of MEST emerged in WAT samples of obese adults compared to controls (31).

Equally controversial data were reported by Gagné-Ouellet et al. (15), which highlighted another DNA methylation site related to GDM at the level of the lipoproteinlipase (LPL) gene. They observed that the different degree of GDM-related methylation within the intergenic region of placental LPL (fetal side) is associated with lower birth weight and BMI before 5 years of age, according to WHO growth charts. Thus, they hypothesized that these epi-variations at the level of LPL on the fetal side of the placenta may compromise fetal growth and fat accumulation in infancy. Hence, these alterations are at the basis of metabolic dysfunctions in later life. However, these remain speculations, in fact whether this association is mediated by epigenetic mechanisms that influence the expression of the LPL gene has yet to be demonstrated.

Epigenetic epidemiology investigation concerned the possible effects on the central nervous system and its neurotransmitters, as well. The study on human placenta by Blazevic et al. (35) suggested that methylation of the gene of the serotonin transporter SLC6A4, one of the main regulators of the homeostasis of this hormone, is sensitive to maternal glycemic alterations during pregnancy. A predominant role of epigenetics, compared to genetics, was evidenced in the regulation of the expression of SLC6A4 in the human placenta. However, further investigations are needed to verify the extent to which these alterations are involved in the long-term GDM-associated effects. Another epigenetic mechanism potentially involved in the effects of GDM on fetal neurodevelopment was studied *in vitro* by Mishra et al. (36). They found that GDM-induced glycemic alterations decrease the expression of the sirtuins-1 gene (SIRT-1), a nutrient sensor, responsible for a decrease in placental transport of DHA through trophoblasts. This mechanism seems to have a central role in the neurodevelopmental dysfunctions observed in children of diabetic mothers (37, 38). A recent meta-analysis supported the hypothesis of the impact of GDM on the neurological development of offspring (39) conducted by the Consortium Pregnancy and Childhood Epigenetics. It analyzed 3,677 mother-infant pairs from seven distinct cohorts, including 317 pairs with GDM. They found a hypo-methylation in cord blood at the level of the OR2L13 promoter, a condition similar to that observed in subjects with autism spectrum disorder. They also discovered a hypo-methylation of CYP2E1, the upregulation of which, in the peripheral blood, is characteristic of subjects with type 1 and type 2 diabetes.

## GDM-correlated complications in offspring

The numerous GDM-related adverse effects are now known despite the still weak evidence of epigenetic epidemiology in this regard. Surely the initial contribution of the Hyperglycemia and Adverse Pregnancy Outcome (HAPO) Study (5) conducted in 15 centers in 9 different countries, was decisive as it definitively established a linear association between short-term adverse effects in pregnancy and poor glycemic regulation. However, to date, the knowledge on the GDM-related offspring complications has significantly expanded to include longer-term effects (40).

### Perinatal, fetal and neonatal complications

One of the best known GDM-related fetal complications is macrosomia, typically defined as a birth weight greater than the 90th percentile for gestational age or a weight >4 kg (41). In fact, there are numerous studies that report this association (5, 42–45), even if the pathophysiological mechanisms underlying this adverse effect have not yet been fully clarified (41). It has been hypothesized that maternal hyperglycemia leads to fetal hyperinsulinemia with consequent weight gain (41). This is because in the presence of maternal glycemic dysregulation, the high circulating glucose levels reach the fetus through the placenta which, however, is not crossed by maternal insulin. Then, in the second trimester of gestation, when the fetal pancreas becomes able to secrete insulin and begins to respond to hyperglycemia and releases insulin autonomously, regardless of glucose stimulation, excessive secretion of this hormone occurs. The fetus is therefore in a highly anabolic condition due to the high concentrations of glucose and hyper-insulinemia which favors weight gain (41). The correlation between GDM and macrosomia already emerged from the HAPO study (5), where an important positive association was found between maternal glycemic levels and birth weight. This correlation was then confirmed by a large retrospective study (43) conducted on 21,000 women. It showed that GDM correlates with a 48% increased risk of fetal macrosomia, a value greater than what emerged in an analysis of the literature conducted until then (15–45%) (41). However, recently, a study by Li et al. based on a multi-racial US population (46), suggested that in the presence of GDM the increase in fetal size compared to the standard, began around 20 weeks of gestation and then become significant only at 28 weeks. These data agreed with the excessive growth of the abdominal circumference found between 20 and 28 weeks of gestation by Sovio et al. (47). This finding highlighted the problem of poor alignment between the need for early intervention timings and standard diagnosis times for GDM, in support of the usefulness of earlier markers. Another retrospective analysis (42) carried out in China, in 2021, on 8,844 women, confirmed the association between GDM and an increased risk of macrosomia, cesarean section and preterm birth, as observed in previous analyzes (43). Furthermore, the latter two events could also be associated with the greater vaginal infection risk in the presence of GDM. This was also found in China in a prospective study in 2015 (48), which showed that in the presence of GDM there was a higher rate of premature rupture of membrane (PROM) and chorioamnionitis. Moreover, this was in agreement with the higher GDM correlated risk of infection reported in the literature (41). Closely associated with the hyperglycemic and hyper-insulinemic state of the newborn of a diabetic mother is hypoglycemia, which is in fact described among the GDM-related adverse neonatal outcomes (41). Indeed, it is the natural consequence of the normal postnatal adaptation to different blood glucose concentrations (49).

The correlation between GDM, perinatal death and congenital anomalies is still controversial, according to a recent review of the literature by Johns et al. (40). These data were then confirmed by an Israeli case-control, retrospective and monocentric study on 526 mothers diagnosed with GDM or pre-gestational diabetes (PGDM) and their children, between 2015 and 2017 (50). Indeed, this investigation revealed that a higher incidence of intrauterine fetal death and congenital anomalies was mainly associated with PGDM, probably also due to the impact of hyperglycemia on embryogenesis during the first 8 weeks of gestation. Other short-term adverse effects associated with the presence of GDM found in the literature (41) are possible neonatal asphyxia, jaundice and kernicterus, neonatal respiratory distress syndrome and birth trauma, including brachial plexus injury and shoulder dystocia, favored by the greater adipose accumulation between the shoulders and the abdominal circumference (47). Polycythemia, hypocalcemia and hypertrophic cardiomyopathy were also found to be GDM-related complications (50, 51). Finally, a significant increase in the thickness of the interventricular septum and of the ventricular wall were found in newborns from mothers with GDM even if in the absence of echocardiographic evidence of congenital heart disease (52).

### Long-term complications

The main long-term consequences on the offspring of mothers with GDM, described in the literature, concern glycemic control, obesity, cardiovascular diseases, neuro-development, neuro-psychiatric morbidity and ophthalmic problems.

Already in 2001, a review of the literature by Dabelea and Pettitt (53), highlighted several scientific evidence regarding the fact that exposure to a hyperglycemic intrauterine environment may be an important risk factor, in addition to genetic predisposition, for the onset of type 2 diabetes (T2DM) in childhood and adolescence. Furthermore, several studies conducted in the following years confirmed these first evidence. Indeed, Dabelea et al. (54), also studied a multiethnic population (African American, Hispanic and non-Hispanic) aged between 10 and 22 years. It emerged that 30.4% of young people with type 2 diabetes were exposed to GDM compared to 6.3% in the unexposed group. It was also found that the association between intrauterine GDM exposure and the onset of type 2 diabetes in offspring is not explained by infant BMI, supporting possible beta-cell dysfunction in offspring (54). Similar results were found in a cohort of 18–27-year-olds with Caucasian prevalence, in which the incidence of type 2 diabetes and pre-diabetes (impaired glucose tolerance or impaired fasting glucose) was 21, 12, 11, and 4%, respectively in the offspring of women with GDM treated with diet therapy, in the children of genetically predisposed women but with normal oral glucose tolerance test (OGTT), in the offspring of mothers with type 1 diabetes and in the children of healthy controls (55). In agreement with these data, Holder et al. showed in a cohort of obese adolescents that 31.1% of subjects with normal glucose tolerance exposed to GDM developed a reduction in glucose tolerance/diabetes in the short follow-up period (2.8 years) compared to 8.6% in the non-exposed group (56). Moreover, in this investigation, they showed an inability of pancreatic beta cells to adequately compensate in response to the decrease in insulin sensitivity in the offspring of mothers with GDM.

The presence of metabolic markers of insulin resistance in the offspring of mothers with GDM has also been documented by several studies (57, 58), as well as the higher incidence of diabetes during adulthood (55).

This is confirmed by a recent (59) study, conducted on the same cohort as the HAPO study, aimed at analyzing the correlation between maternal glycemic values during gestation and the effects on glycemic control of the offspring. It proved that the intrauterine hyperglycemia was significantly associated with glucose and insulin resistance in childhood, regardless of the child's BMI and family history of diabetes. At the same time, Blotsky et al. (60) performed a retrospective cohort study on the population of Quebec and Canada which confirmed the association between GDM and the higher incidence of diabetes during childhood and adolescence. They also highlighted the need for future studies to evaluate the long-term consequences, in terms of severity and negative outcomes, in patients with pediatric diabetes resulting from a maternal history of GDM compared to other patients with childhood or juvenile diabetes.

The presence of a correlation between maternal glucose values and neonatal adiposity was already found in the HAPO Study (61), the results of which seemed to suggest that this relationship could be mediated by the production of fetal insulin. As for obesity and overweight during childhood and adolescence of children of diabetic mothers, although there is numerous scientific evidence regarding the existence of an association between BMI in childhood and GDM (62, 63), it is not yet fully understood how much the influence of maternal pre-pregnancy BMI (58, 64) and being large for gestational age infants (LGA), (58, 65) could contribute. In fact, Boerschmann et al. (60) showed that a combination of genetic predisposition, fetal over-nutrition and lifestyle contribute to the growth during infancy of the offspring of mothers with GDM. In this regard, in 2011 a systematic review and a meta-analysis of the literature showed that maternal diabetes is associated with an increase in the z score of the BMI of the offspring of diabetic mothers (66). However, it is no longer evident after correction for pre-pregnancy maternal BMI (in the limited number of studies where it is reported) (66).

Nevertheless, in the same year, Deierlein et al. (67), analyzed BMI values at the age of 3 and found that fetal exposure to high maternal glucose concentrations in the absence of pre-existing diabetes or with GDM may contribute to the development of overweight/obesity in the offspring, regardless of the maternal pre-pregnancy BMI. Similar results were found by Zhu et al. (68), which highlighted the presence of a significant association between fasting plasma glucose concentrations during pregnancy in women with GDM and the size of offspring at birth and the risk of obesity at 7 years, despite correction for pre-pregnancy maternal BMI values. In contrast, from the multinational cross-sectional study by Zhao et al. (69), on 4,740 children aged 9–11, an association, not completely independent of maternal BMI, was found between GDM and higher probabilities of childhood obesity at 9–11 years. Nonetheless, Tam et al. (62) obtained different results on 970 mother-child pairs of the HAPO Study, 7 years after childbirth. This analysis showed that maternal hyperglycemia in pregnancy is independently associated with the risk of obesity of 7-year-old offspring, exclusively for women. Finally, a large retrospective Chinese population-based study by Chen et al. showed that LGA offspring from mothers with GDM had a higher BMI z score and a higher risk of overweight from 1 to 6 years of age. However, the association was attenuated after correction for pre-pregnancy maternal BMI.

The recent systematic review and meta-analysis by Hammoud et al. (65), revealed a correlation between the presence of GDM and higher blood pressures in the offspring. This is probably at the origin of the greater occurrence of cardiovascular problems (CVD) found in GDM offspring from adolescence to adulthood (70, 71). In fact, a recent 40-year follow-up study on a large cohort of the Danish population that included both pregnant women with GDM and with type 1 and type 2 diabetes, revealed that the offspring of diabetic mothers, particularly those with a past history of CVD or other diabetes-related complications, had increased rates of CVD especially of early onset during the first decades of life (71).

The consequences of GDM on the neuro-development and neuro-psychiatric morbidity of the offspring of diabetic mothers are equally relevant (72). Specifically, the main alterations detected, concern the impairment of language development up to middle childhood (73), the mental and psychomotor development (74) as well as the development of neural systems associated with the recognition of facial expression (75), in the first years of life and the performance of explicit memory at the year of age (76). Moreover, they highlighted Worse offspring school entry assessment scores, intelligence quotient (IQ) and General Certificate of Secondary Education scores (GCSE) (77). Furthermore, in 2016, a cohort study (78), demonstrated that GDM exposure is an independent risk factor for long-term neuro-psychiatric morbidity of the offspring, also highlighting a possible correlation with autism spectrum disorders.

Regarding ophthalmic problems, a recent cohort study by Du et al. based on the Danish population (79), found that maternal diabetes during pregnancy is associated with an increased risk of high refractive error (RE) in the offspring from the neonatal period to adulthood, in all age groups (<3 years, 4–15 years and 16–25 years), especially in children of mothers who have developed diabetes-related complications. These results seem to suggest that the intrauterine ocular impairment induced by maternal diabetes may contribute to an incorrect emmetropization at an early age or to a subnormal refractive accommodation during eye growth in childhood and adulthood. These mechanisms, over time, may further contribute to the high risk of developing elevated RE (79), one of the most common vision disorders that represent the second leading cause of disability globally (80).

## Metabolomics and children of diabetic mothers

A literature review was performed with the following keywords “metabolomics,” “GDM offspring/infants,” or by cross-referencing in already published reviews. The results are summarized in Table 2.

**Table 2:** Metabolomic studies of infants born to women with and without GDM.

**References**	**Sample**	**Bio-specimens**	**Technique**	**Results**	**Clinical significance**
Logan et al. (81)	18 IDM and 12 healthy term control infants	Urine samples	^1^H-NMR	Differences in: glucose, formate, fumarate, succinate and citrate	The urinary metabolome of IDM and term neonates appears to differ and the metabolites involved are associated with the TCA cycle
Dani et al. (82)	30 term IGDMs and 40 controls	Cord serum	^1^H-NMR	↑ Level of pyruvate, histidine, alanine, valine, methionine, arginine, lysine, hypoxanthine, lipoprotein and lipid in GDM babies ↓ Level of glucose	GDM can change neonatal metabolomic profile at birth without affecting the clinical course
Peng et al. (83)	197 Controls and 142 GDM meconium and 96 controls and 81 GDM urinary samples	Meconium and urine samples	^1^H-NMR	14 MECONIUM biomarkers and 3 urinary biomarkers of GDM indicating disruptions of lipid metabolism, amino acid metabolism and purine metabolism	A combined model of 9 meconium biomarkers suggested a great potential in diagnosing GDM-induced disorders
Shokry et al. (84)	200 Mothers and 124 newborns, with an overlap for 119 mother/child pairs PREOBE study cohort	Vein umbilical cord blood samples	LC–MS/MS	↓ Free carnitine, AC, long-chain NEFA, PL, specific Krebs cycle metabolites, and β-oxidation markers	The association between GDM and cord blood metabolites supports the hypothesis of transgenerational cycle of metabolic disorders
Zhao et al. (85)	40 Pregnant women and newborns (22 with GDM and 18 controls)	Maternal fecal samples, neonatal dry blood spots	^1^H-NMR	Lysine, putrescine, guanidinoacetate, hexadecandiodate, negatively correlated with maternal hyperglycemia, contribute to the separation of normal and hyperglycemic neonatal blood metabolomes.	Maternal fecal metabolites contribute to the connections between maternal fecal metabolome and the neonatal blood metabolome
Mansell et al. (86)	912 mother–child pairs in the Barwon Infant Study pre-birth cohort	Maternal serum and at 28 weeks gestation, cord blood serum.	^1^H-NMR	↑ Acetoacetate and 3- hydroxybutyrate	Associations between GDM and higher offspring ketone levels at birth are consistent with maternal ketosis in diabetic pregnancies
Chen et al. (87)	418 mother/neonate pairs (147 women with GDM and 271 controls)	Maternal serum, neonatal meconium	UPLC-QE	Taurine, hypotaurine, pyrimidine, beta-alanine metabolism and bile acid biosynthesis were altered in GDM subjects.	GDM could alter the maternal serum metabolome influencing the neonatal meconium metabolome, highlighting the importance of maternal factors on early-life metabolism.
Herrera-Van Oostdam et al. (88)	26 newborns of GDM mother and 22 controls	Maternal serum, neonatal urine	LC-MS/MS- FIA-MS/MS	Differences in 11 metabolites by univariate analysis. Dysregulation of acylcarnitines, amino acids, and polyamine metabolism by multivariate analysis ↑ (C16:1) and spermine	Metabolic alterations highlight the importance to monitoring the impact of GDM in the early stages of life.

IDM, mothers with diabetes; IGDMs, infants of gestational diabetic mothers; GDM, gestational diabetes mellitus; TCA, *tricarboxylic acid cycle*;

1 H NMR, nuclear magnetic resonance spectroscopy; C16:1, hexadecenoylcarnitine; LC-MS/MS, liquid chromatographic mass spectrometry; FIA-MS/MS, flow injection analysis mass spectrometry; UPLC-QE, ultraperformance liquid chromatography-Q exactive. ↑ represents an increase and ↓ represents a decrease.

The studies regarding the analysis of the metabolomic profile of the offspring of diabetic mothers are quite limited despite their potential usefulness from a preventive point of view. In fact, through the metabolomic investigation, it would seem possible to correlate the qualitative and quantitative changes of metabolites in biological samples of offspring of mothers with previous GDM with a possible development of pathologies, in order to diagnose, prevent or implement early therapeutic strategies (89).

The first data in the literature date back to 2012 from Logan et al. (81). They studied urine samples of newborns from mothers with GDM through a ^1^HNMR analysis, comparing them with a control group, hypothesizing differences in metabolomic profile. The most significant differences that were observed concern the urinary concentrations of glucose, fumarate, formate, citrate and succinate: all involved in the tricarboxylic acid cycle.

In 2014, Dani et al. (82) compared the metabolomic profile of newborns of diabetic mothers with those of healthy mothers in order to evaluate the possible persistence of metabolic alterations despite a close control of the diabetes in pregnancy. They analyzed the cord blood of 30 infants from mothers with GDM and 40 controls, detecting a clear alteration of the metabolomic profile. In fact, lower concentrations of glucose and higher concentrations of pyruvate, histidine, alanine, valine, methionine, arginine, lysine, hypoxanthine, lipoproteins and lipids were detected in children of diabetic mothers. However, they found no significant clinical differences. In 2015, Peng et al. (83) analyzed the metabolomic profile of urine and meconium of 142 newborns from mothers with GDM and 197 controls. Even in this case, the metabolomic profile of the children of diabetic mothers was different from the controls. They found 14 biomarkers in meconium: 10 were endogenous and would seem to be related to gestational diabetes. While, in urine, only 3 metabolites levels were significantly different: uric acid, uridine and estrone. There were also the alterations in lipid, amino acid and purine metabolism in infants from mothers with GDM. In addition, the association between potential biomarkers and related GDM risks were analyzed by a binary logistic regression and an operating characteristic analysis (ROC). Thus, they combined 9 meconium biomarkers in a model which seems to have a great diagnostic potential for the consequences of GDM. Arginosuccinic acid, methyl-adenosine and methyl-guanosine were some of most relevant of those biomarkers.

In 2019, Shokry et al. (84), confirmed the presence of specific related GDM metabolites in the cord blood of children of diabetic mothers. Specifically, the concentrations of free carnitine, acyl-carnitine, long-chain non-esterified fatty acids (NEFA), phospholipids, some intermediates of the Krebs cycle and ß-oxidation (decreased AC2:0/AC16:0 ratios) were lower, supporting possible trans-generational metabolic effects.

Mansell et al. (86) analyzed the metabolomic profile of the cord serum as well, of some subject belonging to the pre-natal cohort of the Barwon Infant Study highlighting that in the presence of GDM there were two distinctive metabolites, vinegar-acetate and 3-hydroxybutyrate, present at higher concentrations. These findings were in agreement with associated GDM ketosis gravidarum, due to lower glucose up-take at the cellular level.

Besides, Zhao et al. (85) found a close correlation between the maternal fecal metabolome and the neonatal blood metabolome. In fact, 4 maternal fecal metabolites, lysine, putrescine, guanidinoacetate and hexadecandiodate, negatively correlated with maternal hyperglycemia, were shown to be responsible for the separation of the metabolome of the neonatal blood of children of diabetic mothers compared to controls. They also showed a correlation between these metabolites and the neonatal metabolome of children suffering from inborn errors of metabolism (IEM) (85). This supports the hypothesis of the important contribution of epigenetic regulation in pathologies with an important genetic basis. In addition, the analysis of metabolic pathways in this study (83), demonstrated a close association between these metabolites and biotin metabolism leading the authors to conclude that biotin metabolism contributes to maternal hyperglycemia as well, and therefore to changes in the neonatal blood metabolome.

Moreover, Chen et al. (87), studied the metabolomic profile of neonatal meconium and that of maternal blood of 418 mother-child pairs from the GDM Mother and Child Study (147 GDM and 271 controls) highlighting a close correlation between the two, supporting the important impact of maternal factors on offspring metabolism. In meconium, an important alteration of the metabolic pathways of taurine, hypo-taurine, pyrimidine and beta-alanine was observed together with variations in the biosynthesis of bile acids.

While, the first work to report quantitative values of concentrations for 101 metabolites measured in the urine of newborns from diabetic mother within the first 24 h of life was performed by. Herrera-Van Oostdam et al. (88). The univariate analysis of these metabolites revealed statistical differences in 11 metabolites, while the multivariate analysis found a different metabolomic profile in neonates of GDM mothers, due to a dysregulation of the metabolism of acyl-carnitines, amino acids and polyamines, together with higher concentrations of hexadecenoyl-carnitine (C16: 1) and spermine, the most discriminant metabolites. The authors pointed out that altered spermine appears to be related to childhood obesity, oxidative stress, circulating leptin values and even possible alterations in the gut microbiota (88).

In this context, Zhao et al. (85) searched for an association between metabolomics and the data on the glycemic response during pregnancy: an analysis of greater proportions, albeit not selective, on GDM. They carried out targeted metabolomic assays on cord blood from European, Afro-Caribbean, Thai and Mexican-American children (400 for each group) whose mothers participated in the HAPO Study (Hyperglycemic and Adverse pregnancy Outcome Study). A meta-analysis of the data collected in the different study cohorts was then carried out, which showed a strong correlation between neonatal and maternal fasting metabolites at 28 weeks of gestation. However, as regards the altered maternal blood sugar, no significant correlations emerged with the amino acid metabolites but rather with a small number of lipid metabolites. Nevertheless, 3-hydroxybutyrate and its carnitine ester at the in the umbilical cord blood were associated with the maternal response to a glucose load (1-h glucose) (90).

On the other hand, there are broader implications in addition to the evaluation of the change in the metabolomic profile of the newborn of a diabetic mother. Indeed, Ott et al. (91) suggest the importance of metabolomic programming by demonstrating that several metabolites, potentially relevant for the development of the disease, are shared between mothers with GDM and their offspring even several years after delivery, regardless of other factors such as BMI or insulin sensitivity. These include carnitine, taurine, creatine, proline, sphingolipid SM (-OH) C14: 1 and glycerophospholipid PC a and C34: 3. These observations led the authors to suppose that this correlation is determined by a shared metabolic pathway rather than merely a transplacental transfer of these metabolites.

## Microbiota in children of diabetic mothers

The importance of the microbiota for the onset and development of metabolic diseases is now recognized by the scientific community (92). Nevertheless, neither the individual components of the bacterial flora with protective or favoring action nor the possible intervention targets have yet been defined (93). During pregnancy, this aspect becomes even more relevant. Indeed, if, on the one hand, the maternal microbiota directly affects pregnancy by influencing the intrauterine environment, the vaginal environment and the risk of preterm birth; on the other hand, it would also seem to affect the nascent microbiota of the offspring, with equally important consequences (94, 95). In fact, it is now established that an alteration of the intestinal microbiota in the early stages of development is related to modifications of the immune system, inflammatory, allergic and metabolic diseases (96–98). Moreover, the fact that the third component of breast milk, unlike all other mammalian species, is represented by oligosaccharides (Human Milk Oligosaccharides, HMOs), specific compounds with a marked prebiotic action, supports the importance of the intestinal bacterial flora from the beginning of life (99). To date, the scientific community seems to agree that maternal health directly influences the intestinal microbiota of the fetus, also through a possible vertical mother-child transmission during pregnancy, albeit still in the absence of certainties on the precise mode of intrauterine microbial acquisition (94, 98, 100, 101). Indeed, the dogma that the human fetal environment is sterile in physiological conditions and that the first microbial colonization of the neonatal intestinal tract begins during birth, both vertically (maternal microbiome) and horizontal (environment) has been questioned (95, 98). Recent studies, through new culture-independent microbiological investigation techniques, have therefore hypothesized that the beginning of the colonization of the fetus by the microbiota begins *in utero*, despite the methodological limitations (95). Specifically, two main transmission routes, still under investigation, have been theorized in healthy pregnancies: a vertical ascension from the vagina and/or urinary tract and a hematogenous pathway through the placenta thanks to a translocation from the digestive tract (buccal cavity and intestine) (98). The detection of the microbial presence in meconium and amniotic fluid is added to further support the hypothesis of colonization *in utero*, although even in this case there is no agreement in the scientific community due to possible methodological limitations and post-natal contaminations (98).

Nowadays, there is a growing number of studies demonstrating an association between GDM and changes in the intestinal bacterial flora both in the mother (18, 102) and in the offspring (18) and specific alterations of the microbiota seem to be found both in the mother and in the progeny. Furthermore, the trend of some of these altered bacterial species appears similar in the mother-child couple, supporting not only a possible form of intergenerational transmission but also the impact of the maternal microbiota on the intestinal colonization process of the offspring (18).

At present, we have found only 5 studies in the literature conducted on the microbiota of the child of a mother with GDM (Table 3), 4 of which looked specifically at the microbial composition of meconium. We used the keywords “microbiota,” “microbiome,” “GDM infants/offspring” together with cross-referenced bibliographical references.

**Table 3:** Studies investigating GDM neonatal microbiome.

**References**	**Patients**	**Specimens**	**Technique**	**Results**	**Clinical significance**
Wang et al. (103)	486 pregnant Chinese women and neonates (GDM and controls)	581 maternal (oral, intestinal and vaginal) and 248 neonatal (oral, pharyngeal, meconium and amniotic fluid) samples	16S rRNA gene and metagenomic sequencing	Pregnant women and neonates microbiota was remarkably altered in GDM Microbial variation concordance between mothers and neonates suffering from GDM	GDM can alter the microbiota of both pregnant women and neonates at birth, showing another form of inheritance and highlighting new prospect for intervention.
Ponzo et al. (104)	29 GDM infants	GDM neonatal fecal samples	16S rRNA gene sequencing	Significantly lower α-diversity in the GDM offspring's microbiota than the corresponding mothers but few Bacteroides and Blautia oligotypes were shared. GDM infants showed higher relative abundance of proinflammatory taxa, a low complexity and a high inter-individual variability. Earlier maternal nutritional habits were more strongly associated with the offspring microbiota. Separation of the infant microbiota according to the type of feeding (breastfeeding vs. formula-feeding).	Many maternal conditions impact on the microbiota composition of GDM offspring whose microbiota showed increased abundance of pro-inflammatory taxa.
Hu et al. (105)	23 newborns stratified by maternal diabetes status (4 pre-gestational type 2 DM, including one mother with dizygotic twins, 5 GDM) and 13 controls	Meconium samples	16S rRNA sequencing, taxonomy assignment and diversity computation	The bacterial content significantly differed by maternal diabetes status, with ↑ alpha diversity of the pre-gestational type 2 DM group than that of no-diabetes or GDM group. No global difference was found between babies delivered vaginally vs. *via* Cesarean-section.	Meconium microbiome of infants born to mothers with the pre-gestational type 2 DM is enriched for the same bacterial taxa as those reported in the fecal microbiome of adult DM patients. The most robust predictor for the meconium microbiota composition was the maternal diabetes status that preceded pregnancy
Lowe et al. (90)	34 full-term and C-sectioned newborns (20 GDM: 15 GDM grade A1 and 5 GDM grade A2 and 14 controls)	Meconium samples	16S rRNA gene sequencing and bionformatics	↓ alpha-diversity, ↑ Proteobacteria. and Actinobacteria and ↓ Bacteroidetes in GDM newborns. Several phyla found in controls were absent in GDM newborns. Bacteria in GDM_A2 newborns did not show variation compared to controls, which might be attributed to the additional intervention by insulin.	There are important implications for the GDM's effects on the gut microbiota of newborns with possible consequences even later in life.
Shokry et al. (84)	418 pregnant women/neonates (147 GDM and 271 controls) neonates	Meconium samples	16S rRNA gene sequencing	Microbial communities were significantly altered in neonates from the GDM mothers with a ↓ alpha-diversity and in the abundance of Firmicutes and Proteobacteria.	GDM impacts the neonatal meconium microbiota

GDM, gestational diabetes mellitus; DM, diebetes mellitus. ↑ represents an increase and ↓ represents a decrease.

Among these, Wang et al. (103), analyzed the intestinal microbiota both of pregnant women with GDM and of their offspring, highlighting a significant alteration of the substantial differences compared to controls. Furthermore, they showed not only a strong correlation between some bacterial species and the oral glucose tolerance test but also the same trend in microbial variations in the mother-child pair, supporting an intergenerational analogy in the changes of the GDM-associated microbiota. Similar results were highlighted by Su et al. (93), which confirmed a correlation between the condition of maternal diabetes and microbial alterations, evidencing a decreased alpha-diversity, with some specific phyla among *Proteobacteria, Firmicutes, Actinobacteria, Bacteroidetes, Chloroflexi, Acidobacteria*, and totally absent *Planctomycetes*. Overall, it was found that offspring from mothers with GDM were characterized by an increase in *Proteobacteria* and *Actinobacteria* phyla and a decrease in Bacteroidetes and at the gender level, a decrease in *Prevotella* and *Lactobacillus* was observed. Indeed, from the correlation analysis, maternal fasting glucose levels were positively associated with a relative abundance of the phylum *Actinobacteria* and the genus *Acinetobacter*, while they showed a negative correlation with the phylum *Bacteroidetes* and the genus *Prevotella*. In this trial, 20 infants from mothers with GDM were enrolled, 15 of them were diagnosed with grade A1 and 5 with grade A2 gestational diabetes. Both groups of diabetic mothers were treated with diet therapy and exercise, however, those with more severe diabetes also received insulin therapy. The children of the latter category of mothers did not show any statistically significant variation in the microbiota compared to the control group. Herrera-Van Oostdam et al. (88) confirmed these data: they found a significant alteration of the microbial communities in meconium of neonates of mother with GDM, highlighting a reduction in alpha-diversity and at the level of phyla, a significant relative abundance of *Firmicutes* and a significant relative decrease of *Proteobacteria*.

However, these results represent a novelty in the scientific literature as the only data present up to this time were different and by Hu et al. (105). This group of researchers analyzed meconium samples from 23 neonates stratified according to maternal diabetes status (4 with pre-gestational diabetes mellitus, 5 with GDM and 13 healthy controls), showing that all samples contained a diverse microbiota, albeit with a lower diversity of species and a considerable variation from sample to sample, regardless of the type of birth (vaginal vs. cesarean). As regards the impact of maternal diabetes, only greater alpha-diversity was detected in the microbiota of the group with pre-gestational diabetes mellitus and the authors were able to conclude that the most robust predictor of the composition microbial of meconium could be the state of pre-pregnancy diabetes.

Only Ponzo et al. (104) analyzed fecal samples between the third and fifth day of life, after the expulsion of the meconium. They highlighted that the main cause of differences found in the neonatal microbiota are determined by the type of breastfeeding (breast or formula), with a greater abundance of *Bifidobacterium* in breastfed infants. However, it was found that some *Bacteroides* and some *Blautia* oligotypes are shared between mothers with GDM and their offspring, in support of a maternal imprinting.

**Figure 1:**
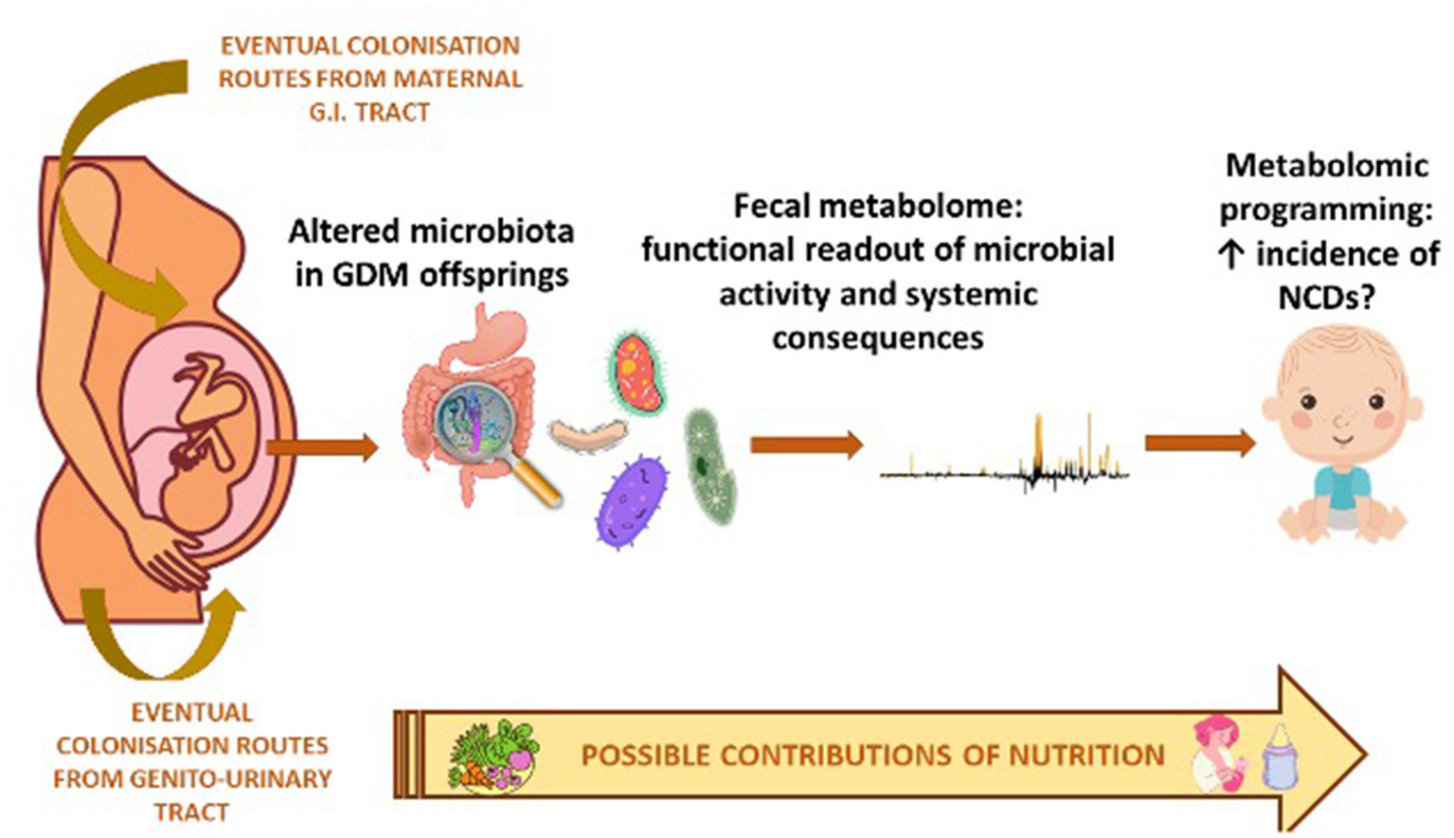
Synergy of action of metabolomic and microbiomic investigation in the offspring of diabetic mother.

The synergy of action of the metabolomic and microbiomic investigation regarding the possible prevention and management of GDM-related complications in offspring, is shown in Figure 1. While, in Table 4 we have summarized the most important biomarkers-target identified in GDM offspring correlated with metabolic alteration through epigenetic, microbiota analysis and metabolomics.

**Table 4:** Principal biomarkers-target identified in GDM offspring correlated with metabolic alteration.

**Techniques**	**principal biomarkers-targets identified**	**Key points**
Metabolomics	↓of specific Krebs cycle metabolites ↓ AC2:0/AC16:0 ratios reflecting a ↓ fatty acid oxidation ↑ argininosuccinic acid: alteration of aminoacids metabolism and specific Krebs cycle intermediates ↓ free carnitine, ↑ (C16:1): alteration in carnitines metabolism ↑ methyladenosine, ↓ methylguanosine: alteration in purine metabolism ↑ spermine: alteration in polyamine metabolism	GDM cord blood metabolites suggest the hypothesis of transgenerational cycle of metabolic disorders concerning aminoacids, purine, lipid, polyamine metabolism and Krebs cycle alterations
Microbiomics	↓ alpha-diversity, ↑ Proteobacteria and Actinobacteria and ↓ Bacteroidetes in GDM newborns ↑ alpha diversity of the pre-gestational type 2 DM group than that of no-diabetes or GDM groups: Bacteroidetes (phyla) and Parabacteriodes (genus) were enriched in the meconium compared to the no-diabetes group Maternal oligosaccharides (from dairy products, cereals, fruit and vegetables) correlated positively with infant *Ruminococcus*, while SFA (from meat and cheese.) were inversely associated with infant *Rikenellaceae* and *Ruminococcus* ↑*Escherichia* and *Parabacteroides* The genera of *Prevotella* and *Lactobacillus* in neonates from mothers treated with insulin did not show any statistical variation	GDM infants' microbiota was remarkably altered, with higher relative abundance of proinflammatory taxa Earlier maternal nutritional habits, health status (pre-gestational type 2 DM) and breastfeeding were more strongly associated with the offspring microbiota GDM newborns' microbiota from mothers treated with insulin administration did not show any statistical variation compared to control
Epigenetics	MEST hypo-methilation in placental tissues and UCB Alterations in fetal placental DNA methylation levels at the LPL gene locus Inverse correlation between PRDM16, BMP7 and PPARGC1A (BAT-related genes) DNA methylation levels and maternal glycemia at the 2nd and 3rd trimester Alteration in LEP and ADIPOQ DNA methylation profile associated with maternal glucose status	Predisposition to obesity, fetal weight and anthropometric profile in children at 5 years of age Variations in DNA methylation in BAT-related genes could mediate the impact of maternal glycemia on cord blood leptin levels

SFA, saturated fatty acids; LEP, leptine; UCB, umbilical cord blood; ADIPOQ, adiponectine. ↑ represents an increase and ↓ represents a decrease.

## Conclusion

In recent years, the numerous advances in the field of metabolomics have clearly highlighted its usefulness in the characterization of various physio-pathological processes (106). Indeed, metabolomics makes it possible to obtain a detailed phenotypic portrait through the dynamic detection of the set of metabolites in cells, tissues and various biological fluids. Therefore, the possibility of assessing the metabolism of the individual with respect to the various pathophysiological stimuli such as the intrauterine environment, diet and drug therapies could represent not only an important prevention tool but also an example of personalized medicine (106). As regards the GDM, the innovation of the use of metabolomic investigation is not only limited to the possibility of an early diagnosis during gestation, even before the clinical manifestation of the disease, but it could also improve the prevention and treatment of GDM-related complications in offspring. The most recent literature suggests how fetal malnutrition and the intrauterine environment can determine a permanent alteration of metabolic processes, influencing the risk of onset of chronic diseases and metabolomics could help both in the identification and management of the complex biological mechanisms that affect future health (107).

Furthermore, in pediatrics, nutritional research is mainly focused on the prevention of the development of pathologies as well as a support in case of illnesses. This is due to the fact that nutrition is a strong modulator of most biological processes that greatly affect the maintenance of health. Therefore, the integration of metabolomics and nutrition to establish individual nutritional phenotypes can help to predict the risk of disease (108), especially in categories of particularly fragile subjects, such as children of diabetic mothers.

Our review has several limitations, mainly due to the fact that the study of the impact of gestational diabetes on offspring through metabolomics and microbiomics is completely new. Thus, there is a lack of standardized and validated quantitative methods for clinical application. Hence, further studies are extremely important. In fact, it might be useful, not only to identify more precisely, through standardized biomarkers within metabolomic databases, the particular altered metabolic pathways, but also to assess the extent of metabolomic programming. Thus, the usefulness of metabolomics is that it could provides the detection of metabolites, potentially relevant for the development of diabetes-related complications, shared in the offspring of mothers with GDM both at birth and several years after delivery. This would allow us to understand more precisely the consequences of GDM on offspring that could originate not only from a transplacental transfer of certain metabolites but also be the result of a shared metabolic path.

In this context, the intestinal microbiota plays a very important role as it is the main responsible for the fecal metabolome which in turn provides a functional portrait of the microbial activity and can be used as an intermediate phenotype that mediates host-microorganisms interactions (109).

It has in fact been shown through the fecal metabolome, or secondary metabolites of microbial origin that the intestinal microbiota exerts a large effect on the biochemistry of the host (110). Therefore, it is clear that any disturbance in the composition of the intestinal microbiota can have an important impact on the health of the host through the modification of the intestinal metabolic profile (110).

Furthermore, such extensive and detailed monitoring of the metabolomic profile and microbiota would allow not only to improve the current knowledge on the overall metabolic structure of the child of a diabetic mother, but could have very important clinical implications. In fact, it would be possible to implement an effective lifestyle intervention in subjects “predestined” for the development of metabolic pathologies, preventing or delaying the pathological manifestation. This would also postpone the complications related to it, a necessary condition for controlling the enormous clinical, social and economic burden of this disease, providing a new direction in terms of early prevention and treatment of GDM. Finally, it would be desirable to manage any alterations detected also through a “personalized nutrition” in perfect harmony with the precise phenotype.

Therefore, it clearly emerges the need for the integration of multi-omics (metabolomics and microbiomics) datasets from different populations, as demonstrated by other authors (111).

## Author contributions

AD wrote and revised the manuscript and original idea for this manuscript. CT and AB wrote the manuscript and revised the literature. RP and VF revised the manuscript. All authors have read and accepted the final version of this manuscript.

## Conflict of interest

The authors declare that the research was conducted in the absence of any commercial or financial relationships that could be construed as a potential conflict of interest.

## Publisher's note

All claims expressed in this article are solely those of the authors and do not necessarily represent those of their affiliated organizations, or those of the publisher, the editors and the reviewers. Any product that may be evaluated in this article, or claim that may be made by its manufacturer, is not guaranteed or endorsed by the publisher.
